# Centralized Consensus Hemagglutinin Genes Induce Protective Immunity against H1, H3 and H5 Influenza Viruses

**DOI:** 10.1371/journal.pone.0140702

**Published:** 2015-10-15

**Authors:** Richard J. Webby, Eric A. Weaver

**Affiliations:** 1 Department of Infectious Diseases, St. Jude Children’s Research Hospital, Memphis, TN, United States of America; 2 School of Biological Sciences and the Nebraska Center for Virology, University of Nebraska, Lincoln, United States of America; Instituto Butantan, BRAZIL

## Abstract

With the exception of the live attenuated influenza vaccine there have been no substantial changes in influenza vaccine strategies since the 1940’s. Here we report an alternative vaccine approach that uses Adenovirus-vectored centralized hemagglutinin (HA) genes as vaccine antigens. Consensus H1-Con, H3-Con and H5-Con HA genes were computationally derived. Mice were immunized with Ad vaccines expressing the centralized genes individually. Groups of mice were vaccinated with 1 X 10^10^, 5 X 10^7^ and 1 X 10^7^ virus particles per mouse to represent high, intermediate and low doses, respectively. 100% of the mice that were vaccinated with the high dose vaccine were protected from heterologous lethal challenges within each subtype. In addition to 100% survival, there were no signs of weight loss and disease in 7 out of 8 groups of high dose vaccinated mice. Lower doses of vaccine showed a reduction of protection in a dose-dependent manner. However, even the lowest dose of vaccine provided significant levels of protection against the divergent influenza strains, especially considering the stringency of the challenge virus. In addition, we found that all doses of H5-Con vaccine were capable of providing complete protection against mortality when challenged with lethal doses of all 3 H5N1 influenza strains. This data demonstrates that centralized H1-Con, H3-Con and H5-Con genes can be effectively used to completely protect mice against many diverse strains of influenza. Therefore, we believe that these Ad-vectored centralized genes could be easily translated into new human vaccines.

## Introduction

Influenza A virus infections impose a significant burden on our healthcare systems. The influenza pandemic of 1918–1919 infected ~25% of the world’s population. Studies have estimated that the mortality rate of this pandemic was greater than 2.5% [1]. The high mortality rate of this pandemic and the widespread global infection rate resulted in 20–100 million deaths [2]. Similarly, the 2009 Swine flu pandemic infected 24% of the global population. However, this pandemic virus had an estimated mortality rate of 0.02% [3]. In the United States of America, the 2009 H1N1 swine flu pandemic resulted in ~68 million infections and 274,304 hospitalizations [4]. The 2009 swine flu pandemic was an ominous reminder of our susceptibility to infectious diseases. Even with our current global monitoring, antiviral drugs and advanced vaccine technology, the pandemic was unstoppable.

A universal influenza vaccine has been the ultimate goal for vaccine researchers. Several strategies have been proposed to expand the breadth of immunity against divergent strains of influenza. The use of conserved genes such as the nucleoprotein (NP) and matrix (M) have shown the potential to induce cross-reactive T cell immunity against divergent influenza strains [5–8]. Studies have shown that antibodies directed to the stalk region of HA induce broadly reactive and neutralizing antibodies in humans and mice [9–11]. An alternative approach to making broadly-reactive influenza vaccines has been through the use of centralized HA genes. A centralized gene is a gene that localizes to the center of a phylogenetic tree. These genes can be made by using computer models that predict the ancestral, COT (center of tree), mosaic or consensus sequences. In these studies we have used the consensus method to create our centralized HA genes. Much of this work stems from early studies using consensus genes as candidate group M Env HIV vaccines [12–15]. In this approach, the vaccine gene is computationally designed so that it localizes to the central node of a phylogentic tree. These centralized sequences have the advantage over wildtype genes in that they are genetically equidistant to all circulating wildtype strains. Therefore, in the case of vaccine mismatch, they are likely to be more closely related to the challenge strain than a wildtype strain. This approach has been investigated for use in the construction of centralized HA genes capable of inducing protection against H5N1 influenza strains [16–19]. Our studies on centralized H1-Con genes showed that, in the case of vaccine mismatch, the centralized consensus gene induced better anti-heterosubtypic immunity against divergent influenza strain as compared to the wildtype genes. In all vaccine mismatch examples the consensus H1-Con vaccinated mice had the greatest level of protection against lethal influenza virus challenges and showed reduced disease burdens and increase survival rates as compared to the wildtype vaccine groups [20].

In this study we have applied the centralized gene vaccine approach to three relevant human influenza virus HA genes, H1, H3, and H5. The centralized genes were incorporated into replication-defective Adenovirus (Ad) viral vector systems and tested to protect against a wide panel of divergent H1, H3 and H5 influenza isolates. We found that *in vitro* and *in vivo* analyses indicate that the Ad-vectored centralized vaccines can provide high levels of protection against a wide array of divergent influenza strains. We feel that combining the robust gene delivery of the Ad vaccine system with the use of centralized genes may provide a foundation of immunity that is capable of providing high levels of cross-protective immunity against many, if not all, human influenza strains.

## Materials and Methods

### Centralized Gene Construction

Centralized H3-Con and H5-Con HA genes were created using human influenza virus sequences created as previously described for the H1-Con gene [20]. Briefly, phylogenetic trees were created and representative sequences of each major branch were selected. This is done to circumvent bias in the database of high throughput sequences that are uploaded through large-scale studies. Eliminating database bias and redundant sequences allows for the creation of centralized genes using the most common amino acid at each position. Selected full-length H3 and H5 HA sequences were downloaded from Genbank and aligned using ClustalX v2.1. The following 26 sequences were used to create the H3 consensus HA gene: (Accession: AY032978, CY002136, AF382328, CY000584, CY000241, CY000569, CY000017, CY000137, CY002904, CY002072, DQ249261, CY002056, CY000609, AB019357, AJ252131, CY002088, CY003512, CY003064, M55059, V01103, AJ289703, CY002496, CY002744, M54895, CY006044, X05907). The following 21 sequences were used to create the H5 consensus HA gene: (Accession: ISDN38262, AY575869, AY555153, ISDN40341, ISDN119678, AB239125, AY555150, ISDN110940, AY651335, AY651334, ISDN118371, ISDN121986, ISDN117777, ISDN117778, ISDN49460, AJ867074, AY679514, AF084532, AF084279, AF084280, AF046097)

All aligned sequences were then inspected manually to correct for apparent mistakes. Positions containing gaps or ambiguously aligned positions were removed from the datasets. The amino acid consensus sequences were generated from this alignment by using the most common amino acid at each position. The 566, 566 and 568 amino acid consensus sequences were named H1-Con, H3-Con, and H5-Con, respectively. The polybasic connecting peptide region was not included in the construction of the centralized H5-Con gene. The HA genes were codon-optimized for mammalian expression and synthesized by Genscript. Inc.

The centralized genes created by this approach retain critical functional domains such as the secretory signal, polybasic connecting peptide, cleavage and fusion sites, transmembrane domains and cytoplasmic tail. The ability of the consensus genes to hemagglutinate cRBCs indicating that they posses the receptor binding domains and transmembrane domains of a functional influenza HA gene is shown (S1 Fig). All 3 consensus proteins have a predicted transmembrane domain similar to wildtype HA genes (S2 Fig). A multi-sequence alignment of all 3 consensus genes is shown (S3 Fig). In order to determine if the consensus genes could produce infectious virions, non condon-optimized consensus genes were made by performing a BLASTP search and identifying the most closely related HA strain. The closely related strains were then modified to have the consensus amino acid sequence using influenza-biased codon usage. The 5’ and 3’ untranslated regions (UTRs) from the A/PR/8/34 strain were incorporated onto the ends of the consensus genes. All 3 genes were then cloned into the pHW2000 plasmid as previously described [21]. The individual consensus genes were cotransfected along with the remaining 7-plasmid set from strain A/PR/8/34 into a 293/MDCK cell mixture. The supernatant from the transfected cells was used to inoculate 10–11 day old embryonated eggs and harvested after 72 hrs of incubation. Cells transfected with all 8 of the A/PR/8/34 control plasmids produced high titer stocks of influenza virus as detected by hemagglutination assay. However, none of the 3 consensus genes were able to produce infectious virions. We believe that there may be problems associated with the 5’ and 3’ UTRs that are having a negative effect on the rescue of infectious virus. We are exploring ways to identify UTRs that will result in a functional positive and negative sense HA RNA segments.

### Adenovirus Vaccines

**Fig 1:**
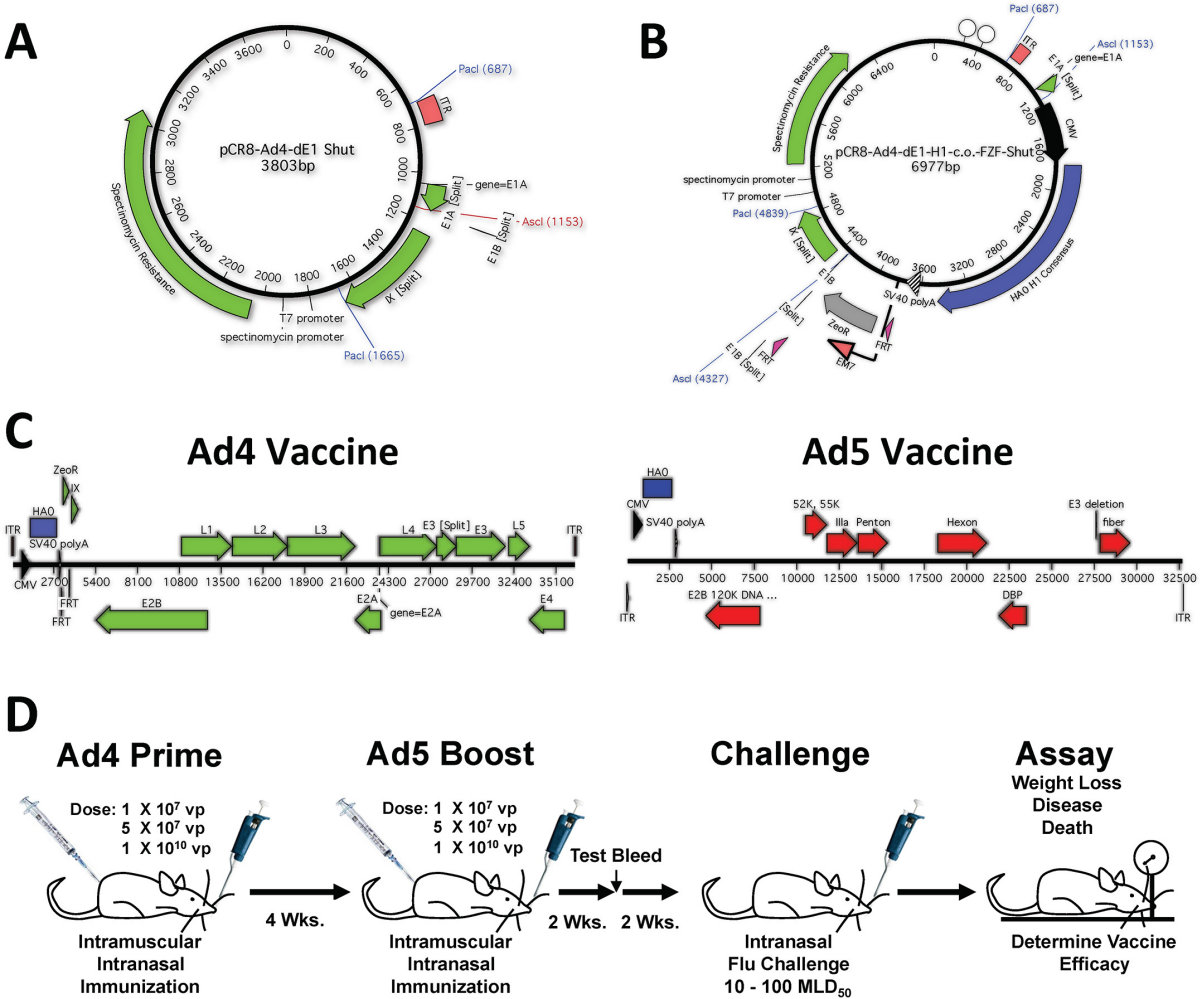
Vaccine Design and Immunization Schedule. A schematic representation of the Ad4 shuttle plasmid is shown (A). The shuttle plasmid containing the CMV-HA-PolyA expression cassette is shown (B). The two Adenoviral types and the location of the HA expression transgenes used as vaccine vectors are shown (C). The Ad4 vaccine had an E1 deletion whereas the Ad5 vaccine had E1 and E3 deletions. The HA transgene expression cassette was recombined in place of the E1 genes in both vaccine vectors. Mice were immunized with the vaccine vectors using three different doses (D). The mice were primed with the Ad4 vaccine vector and boosted 4 weeks later with the Ad5 vaccine vector. Test bleeds were taken 2 weeks post-boosting and the mice were challenged with lethal doses of influenza 4 weeks post-boosting. The mice were monitored for weight loss, disease and death. Mice that lost ≥25% of the day 0 body weight were humanely euthanized.

In order to provide optimal boosting we designed the Adenovirus (Ad) vaccine vectors based on two distinct Ad types 4 and 5 (Ad4 and Ad5, respectively). We have previously described the use of Ad4 vaccine vectors as influenza vaccines. However, these vectors were replication-competent (RC) E3 deleted vectors [22]. In this study, we have utilized E1 deleted replication-defective (RD) Ad vaccines. The RD Ad4 vaccines were constructed similarly to the previously reported RC vectors. However, the E1 genes were deleted and replaced with an expression cassette containing the HA-Con genes. The E1 shuttle consisted of the left ITR fused to the pIX gene with a unique AscI restriction site incorporated between the Ad4 regions (Fig 1A). The Ad4 homologous regions were flanked by PacI restriction sites. A CMV-HA-Con-polyA expression cassette was fused to a FRT-Zeocin-FRT using overlapping PCR with flanking AscI sites. The expression cassette was then cloned into the E1 shuttle plasmid to create the final shuttle vector (Fig 1B). The E1 shuttle plasmid was linearized by digestion with PacI and cotransformed with the complete Ad4 genome plasmid in BJ5183 cells. Recombinant plasmids were selected on Kanamycin/Zeocin LB plates. The recombined plasmid was linearized to release the recombinant Ad4 genomic DNA. The linearized DNA was transfected into 293 cells using Polyfect (Invitrogen). The rescued viruses were serially amplified *in vitro*. The first generation RD (E1/E3 deleted) Ad5 vectors were constructed using the Ad-Easy system in 293A cells as described in [23]. Ultimately, three Ad4 and three Ad5 vaccines were constructed that expressed the three H1-Con, H3-Con and H5-Con consensus genes (Fig 1C). All adenoviruses were purified by banding using two sequential CsCl ultracentrifugation and virus particles (vp) were quantitated by OD260.

### Ad Vaccine Immunization

Groups of 5 mice were anesthetized i.p. with ketamine (140 mg/kg)/xylazine (5.55 mg/kg). Using various doses of the Ad4 vaccines the mice were primed with the consensus HA genes. The mice were boosted 4 weeks after priming with the Ad5 HA-con expressing vaccines (Fig 1D). The Ad vaccines were delivered intramuscularly (i.m.) in a total volume of 50 μl delivered in two 25 μl injections in the quadriceps. Two weeks after boosting, submandibular test bleeds were performed and sera were separated using BD serum separator tubes. The mice were immunized with the individual Ad vaccines at doses of 1 X 10^7^ virus particles (vp)/mouse, 5 X 10^7^ vp/mouse and 1 X 10^10^ vp/mouse.

### Influenza Viruses

Influenza virus H1N1 A/PR/8/34 (PR/34) was obtained from ATCC (VR95). Influenza viruses BEI NR-3170 H1N1 A/Fort Monmouth/1/47 (FM/47), BEI-2759 H1N1 A/WS/33 (WS/33), BEI-NR13663 H1N1 A/California/07/09 (CA/09), BEI NR-3604 H3N1 A/Texas/1/77 (TX/77), BEI NR-3502 H3N2 A/Mississippi/1/85 (MS/85), BEI NR-3483 H3N2 A/Aichi/2/68 (Aichi/68), and BEI NR-12283 H3N2 A/Brisbane/10/2007 (Bris/07) were obtained from the Biodefense and Emerging Infections Research Resources Repository. H5N1 A/BH Goose/Qinghai/A/05 (Goose/05), H5N1 A/Vietnam/1203/04 (Viet/04), and H5N1 A/Japanese White Eye/Hong Kong/1038/2006 (White Eye/06) were received from the WHO GISRS network. The H3 and H5 influenza viruses used in the *in vivo* studies were mouse-adapted through serial lung passage as previously described [24]. All of the viruses were passaged one time in SPF embryonated eggs and the chorioallantoic fluid was stored at -80°C. The influenza virus stocks were titered in BALB/c mice to determine their 50% mouse lethal dose (MLD_50_).

### Animals

Female BALB/c mice (6–8 weeks old) were purchased from Charles River Laboratories (Wilmington, Massachusetts, USA) and housed in the Mayo Clinic Animal Facility under the Association for Assessment and Accreditation of Laboratory Animal Care (AALAC) guidelines with animal use protocols approved by the corresponding the Mayo Clinic Institutional Animal Care and Use Committee (IACUC protocol No. A64612). All animal experiments were carried out according to the provisions of the Animal Welfare Act, PHS Animal Welfare Policy, the principles of the NIH Guide for the Care and Use of Laboratory Animals, and the policies and procedures of Mayo Clinic.

### Hemaglutination Inhibition (HI) Assay

Serum was collected using Becton Dickinson microtainer tubes with serum separator and heat-inactivated at 56°C for 1h. Starting at a dilution of 1:5 sera were diluted two-fold in 50 μl of DPBS in a 96-well, nonsterile, nontissue culture–treated, round bottom microtiter plate. Four HAU of influenza virus in 50 ul was added to the diluted sera and incubated at room temperature (RT) for 1 hr. After incubation, 50 μl of a 1% chicken RBC solution was added and incubated at RT for 1 hr. The HI titer was determined to be the highest serum dilution to inhibit hemagglutination.

### Lethal Influenza Challenge

Four weeks after the boosting immunization the mice were anesthetized i.p. with ketamine (140 mg/kg)/xylazine (5.55 mg/kg). The mice were weighed for baseline measurements and challenged intranasally with 100MLD_50_ of influenza A/PR/8/34, A/FM/1/47 virus, A/WS/33 virus, A/TX/1/77, A/Vietnam/04, A/BH Goose/Qinghai/A/05, A/Japanese White Eye/Hong Kong/1038/2006 virus. Mice were challenged with 10MLD_50_ of A/Mississippi/1/85. The mice were placed on their backs and 10 μl of influenza virus was pipetted into each nare in a total volume of 20 μl. The mice were then weighed and monitored daily for signs of disease. Mice were humanely euthanized if their body weight dropped ≥25% of baseline weights.

## Results

### HI Titers after Monovalent Consensus HA Vaccination

**Fig 2:**
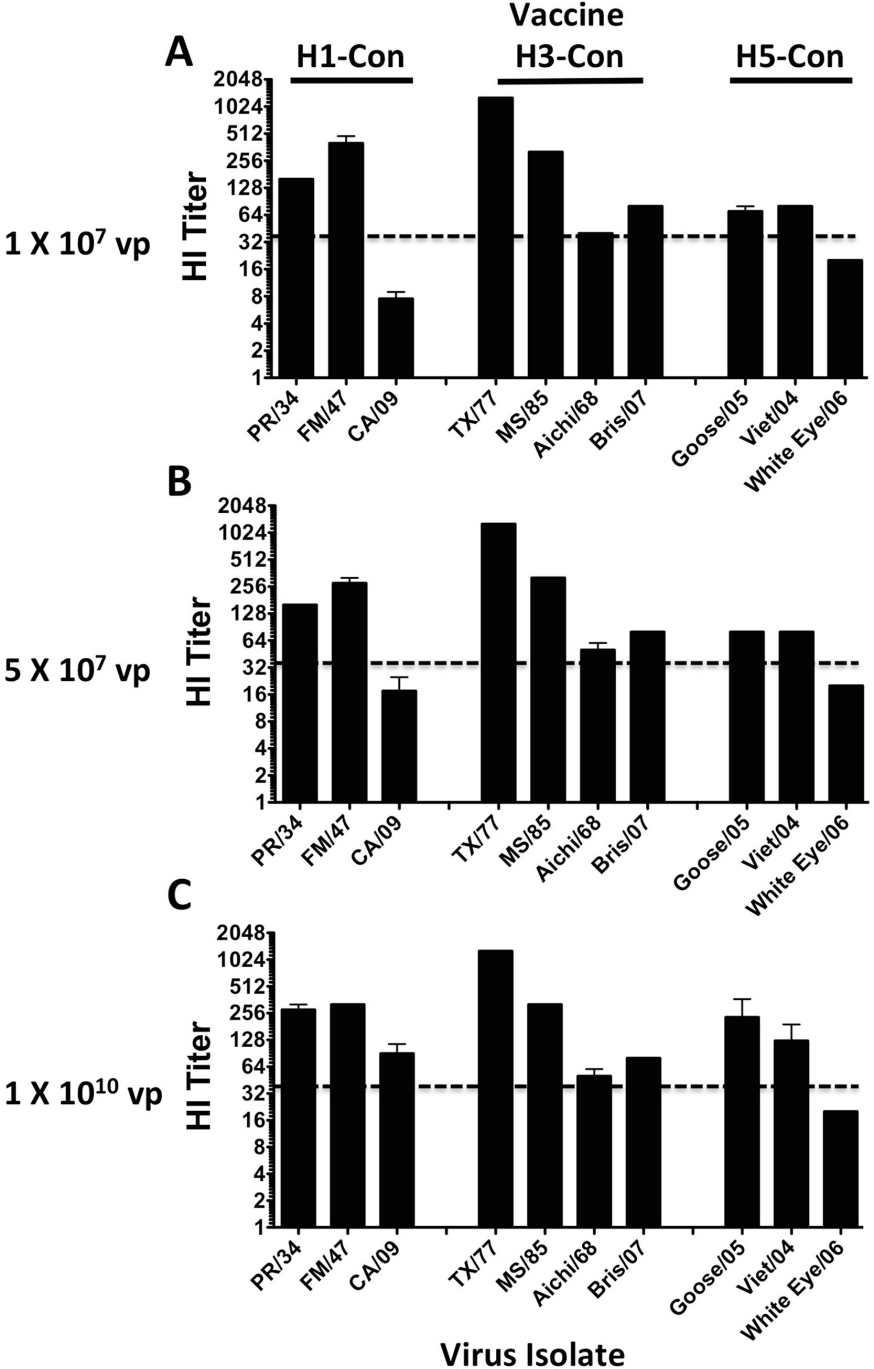
Hemagglutination Inhibition Assays after HA Vaccination. The HI titers were determined in mice immunized with Ad vaccines expressing either the H1-Con, H3-Con or H5-Con consensus HA gene. Ad vaccines were given at 3 different doses. The HI titers induced by the lowest dose of vaccine, 1 X 10^7^ vp/mouse are shown (A). A low intermediate dose vaccine of 5 X 10^7^ vp/mouse is shown (B). A high dose vaccine of 1 X 10^10^ vp/mouse is shown (C). Mice immunized with Ad vaccines expressing the H1-Con gene were assayed for HI antibodies against influenza A/Puerto Rico/8/34 (H1N1), A/Fort Monmouth/1/47 (H1N1) and A/California/04/09 (H1N1). Mice immunized with Ad vaccines expressing the H3-Con gene were assayed for HI antibodies against influenza A/Texas/1/77 (H3N1), A/Mississippi/1/85 (H3N2) and A/Aichi/2/68 (H3N2). Mice immunized with Ad vaccines expressing the H5-Con gene were assayed for HI antibodies against influenza A/Vietnam/1203/04 (H5N1), A/Japanese White Eye/Hong Kong/1038/2006 (H5N1) and A/BH Goose/Qinghai/A/05 (H5N1). The dotted line represents a theoretical titer indicative of protection.

In order to determine if the consensus genes could induce anti-influenza immunity mice were primed and boosted with Ad vaccines expressing only one gene. The HI titers increased in a dose response manner as the amount of vaccine increased. In general, an HI titer of 40 (expressed as the reciprocal of the highest dilution to inhibit hemagglutination) or greater strongly correlates with a protective immune response when evaluating inactivated influenza vaccines in adults [25, 26]. The H1-Con gene induced HI titers greater than 40 for both PR/34 and FM/47 viruses at all vaccine doses. However, only the highest dose of 1 X10^10^ vp was capable of inducing a protective HI titer against the CA/09 virus (Fig 2C). The H3-Con gene induced HI titers ≥40 against all four H3 influenza strains at all 3 vaccine doses (Fig 2). The H5-Con gene induced HI titers ≥40 against both Goose/05 and Viet/04 influenza strains. However, the HI titer never reached 40, even at the highest dose, against the White Eye/06 strain (Fig 2C).

### Protection against Lethal H1N1 Influenza Virus by a Centralized H1-Con Vaccine

**Fig 3:**
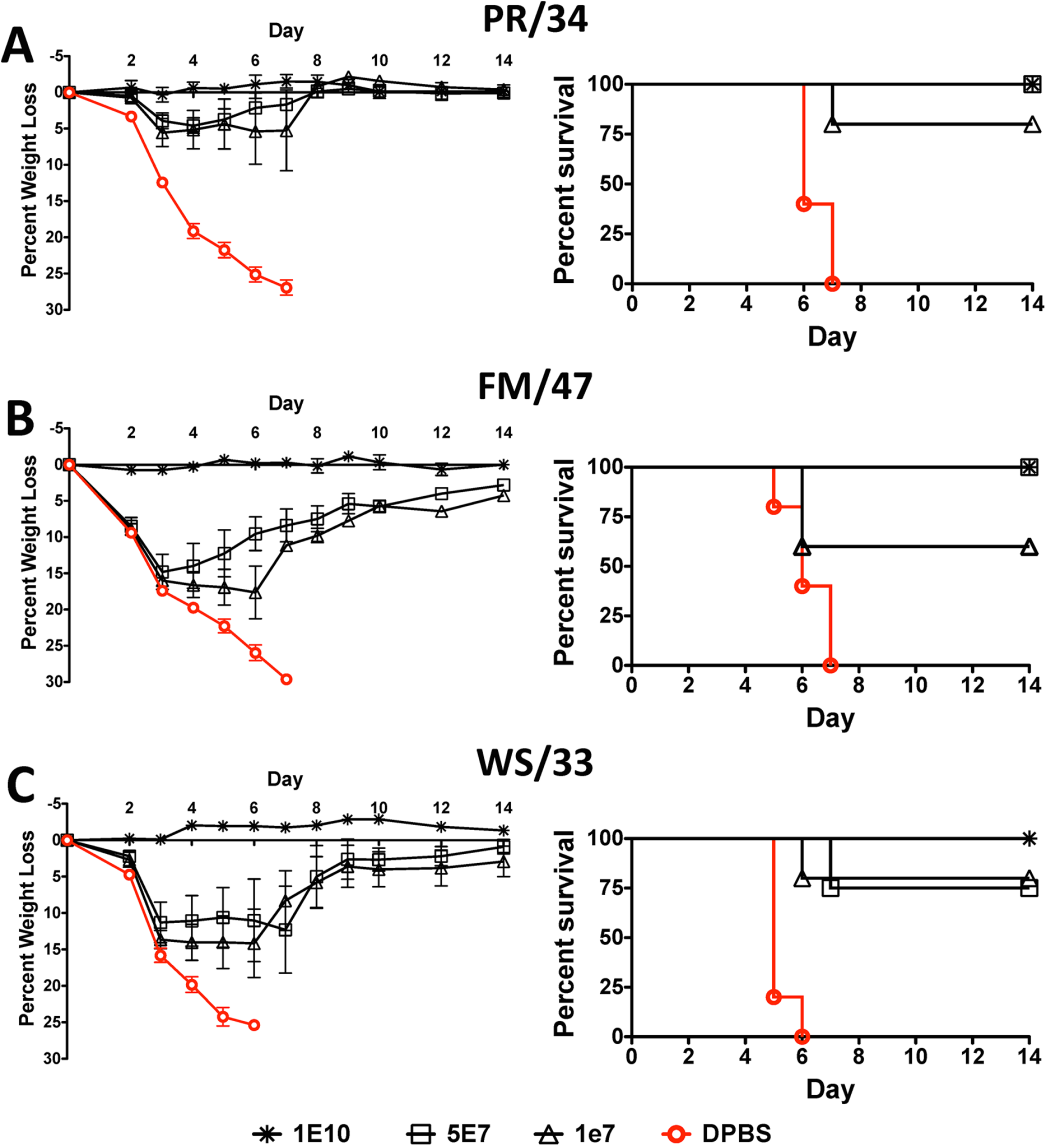
Protection Against Lethal H1N1 Influenza Virus by a Centralized H1-Con Vaccine. Mice were immunized and boosted with various doses of Ad4 and Ad5 expressing the H1-Con consensus HA gene. Four weeks post-boosting the mice were challenged with 100 MLD_50_ of mouse-adapted influenza A/Puerto Rico/8/34 (A), A/Fort Monmouth/1/47 (B), or A/WS/33 (C). The mice were monitored for weight loss, disease and survival. Mice that lost ≥25% of their baseline weight were humanely euthanized.

Mice vaccinated with the highest dose of H1-Con vaccine were completely protected against 3 divergent lethal H1N1 influenza strains (Fig 3). The highest vaccine dose (1 X 10^10^ vp) was capable of providing complete protection from morbidity where there was no weight loss or signs of disease and 100% survival against lethal A/PR/8/34, A/FM/1/47 and A/WS/33 influenza virus challenges. Mice vaccinated with a lower dose of 5 X 10^7^ vp had 100%, 100% and 75% survival against lethal A/PR/8/34, A/FM/1/47 and A/WS/33 influenza virus challenges, respectively. The lowest dose of vaccine, 1 X 10^7^ vp, provided 80%, 60% and 80% survival against lethal A/PR/8/34, A/FM/1/47 and A/WS/33 influenza virus challenges, respectively (Fig 3). Although the lower doses of vaccine did not completely protect against weight loss, the vaccinated mice showed reduced disease as compared to control mice and began to recover quickly with 4 days of showing signs of disease. We tested the ability of the H1-Con vaccine to protect against the 2009 swine flu virus (A/CA/04/09) in a previous study [20]. A single immunization of Ad5 expressing the H1-Con gene resulted in only 80% survival at a dose of 1 X 10^10^ vp/mouse against lethal A/CA/04/09 influenza virus. However, this study only used a single immunization. A prime/boost strategy such as the one included in this study may have resulted in much higher survival rates.

### Protection against Lethal H3N1 and H3N2 Influenza Virus by a Centralized H3-Con Vaccine

**Fig 4:**
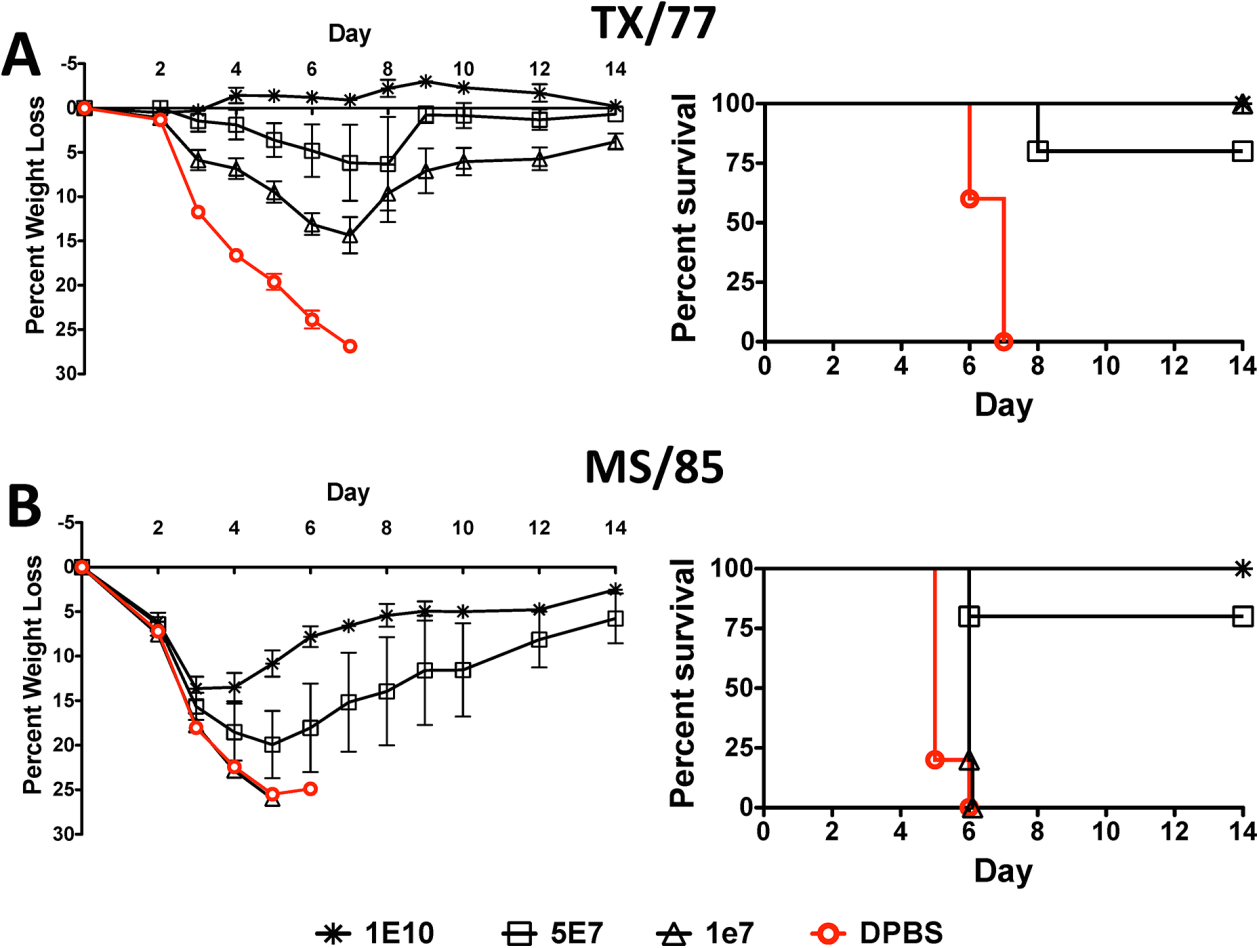
Protection Against Lethal H3N1 and H3N2 Influenza Virus by a Centralized H3-Con Vaccine. Mice were immunized and boosted with various doses of Ad4 and Ad5 expressing the H3-Con consensus HA gene. Four weeks post-boosting the mice were challenged with 100 MLD_50_ of mouse-adapted influenza A/Texas/1/77 (A) or 10 MLD_50_ of A/Mississippi/1/85 (B). The mice were monitored for weight loss, disease and survival. Mice that lost ≥25% of their baseline weight were humanely euthanized.

Mice vaccinated with the highest dose of the H3-Con vaccine were protected against A/TX/1/77 and A/MS/85 lethal influenza challenges. A dose of 1 X 10^10^ vp of the H3-Con gene resulted in 100% survival against both H3 influenza strains (Fig 4). However, this vaccine only eliminated signs of disease in mice challenged with A/TX/1/77 and not A/MS/85 (Fig 4). A vaccine dose of 5 X 10^7^ vp protected 80% of mice against both lethal influenza strains. The lowest dose of vaccine, 1 X 10^7^ vp, was capable of protecting mice against A/TX/1/77 mortality, but not against A/MS/85.

### Protection against Lethal H5N1 Influenza Virus by a Centralized H5-Con Vaccine

**Fig 5:**
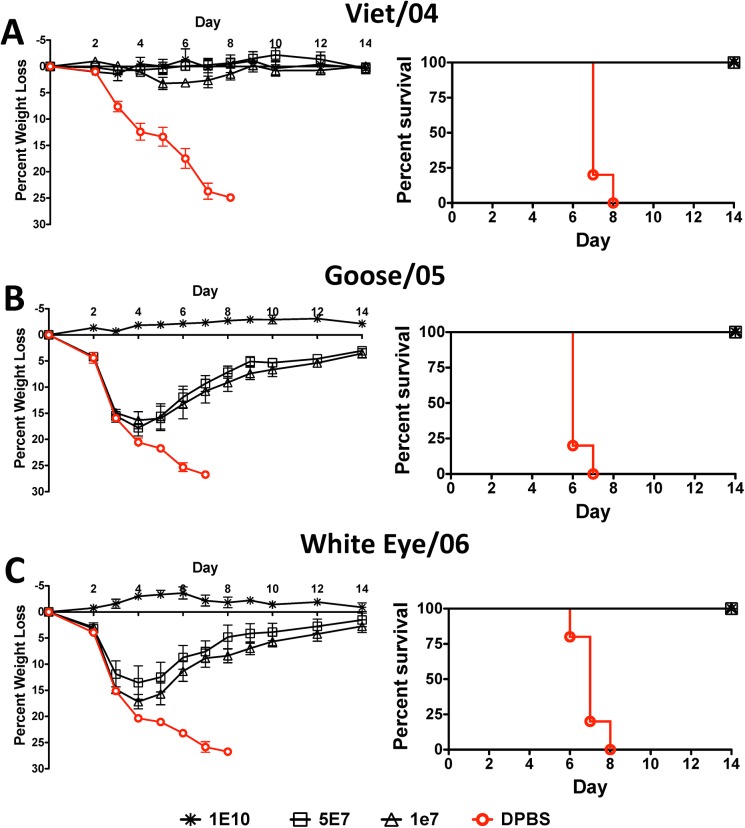
Protection Against Lethal H5N1 Influenza Virus by a Centralized H5-Con Vaccine. Mice were immunized and boosted with various doses of Ad4 and Ad5 expressing the H5-Con consensus HA gene. Four weeks post-boosting the mice were challenged with 100 MLD_50_ of mouse-adapted influenza A/Vietnam/1203/04 (A), A/BH Goose/Qinghai/A/05 (B) and A/Japanese White Eye/Hong Kong/1038/2006 (C). The mice were monitored for weight loss, disease and survival. Mice that lost ≥25% of their baseline weight were humanely euthanized.

Mice vaccinated with the H5-Con vaccine were protected against three divergent H5N1 influenza strains. The highest dose of vaccine resulted in complete protection from morbidity and mortality (Fig 5). The lower doses of vaccine also completely protected mice from the A/VN/04 lethal influenza challenge (Fig 5A). Although the lower doses of vaccine were associated with disease and weight loss when challenged with the A/BH Goose/Qinhai/A/05 and A/ Japanese White Eye/Hong Kong/1036/06, all vaccinated mice survived the lethal challenge (Fig 5B and 5C). In general, the vaccinated mice showed signs of recovery and weight gain within 4 days of the lethal influenza challenge.

## Discussion

There is little dispute that when the vaccine and the challenge virus are identical, protection will be optimal. Previously we showed that immunization with a H1 HA antigen that was homologous to the challenge virus always induced greater levels of protection [20]. It is very likely that we would see the same results for the H3 and H5 HA antigens. A study comparing the consensus genes to the wildtype genes for all 3 HA subtypes would require the construction of many control vaccines and is beyond the scope of these initial studies. However, when there is a vaccine mismatch, the CDC states “it’s important to remember that even when the viruses are not closely matched, the vaccine can still protect many people and prevent flu-related complications against different but related influenza viruses [27].” Therefore, the more closely matched the vaccine formulation is to the infecting virus; the more likely there will be cross-protective immunity. Centralized HA genes localize to the center of the phylogenetic tree. This is a simple, yet effective, way to reduce the genetic distance to all known wildtype influenza strains within each subtype. In the case of a pandemic where there is an antigenic shift and the vaccine strain is significantly different from the pandemic strain, it is possible that a consensus antigen would provide better protection. However, in the case of small antigenic drift endemics a wildtype HA antigen may provide greater levels of immunity. Therefore, the consensus vaccine antigens could be used as a fourth or fifth antigen in the vaccine formulation as a supplement in cases where there is substantial mismatch. The consensus genes were incorporated into Adenovirus types 4 and 5 for evaluation as influenza vaccine vectors.

A key correlate of vaccine efficacy is the HI assay where a titer 40 indicates a 50% reduction in the risk of an influenza infection [28]. We were pleased to see HI titers above 40 in mice vaccinated with the lowest dose of vaccine. Some of the titers were significantly above 40. However, the HI titers are not always correlative with protection. The HI titers against the H3N2 MS/85 strain are well above the predicted level of protection at all vaccine doses. In contrast, only the highest vaccine dose was capable of inducing protection and providing 100% survival against a lethal MS/85 virus challenge. In fact, even the highest dose of Ad-H3 vaccine was not able to prevent disease and weight loss in these mice. Viral clearance was most likely due to a combination of both humoral and cellular immunity. Also in contrast to the HI immune correlate is the fact that the HI titer against the H5N1 White Eye/06 strain never reaches the 40 threshold and yet the vaccinated mice are protected at all doses. The H1-Con vaccine only induced protective HI titers against the CA/09 strain at the highest vaccine dose. In a previous study using the H1-Con centralized gene, we only observed 80% survival in mice immunized with 1 X 10^10^ vp/mouse when challenged with 70 MLD_50_ of influenza CA/09. The primary difference between the studies is that we use an Ad4-H1-Con prime and an Ad5-H1-Con boost. It is likely that the highest dose of Ad-H1-Con vaccine could protect 100% of vaccinated mice, however we did not include in vivo studies with this strain, only the immune correlates.

The ability of the centralized genes to induce intraserotype cross-protective immunity appears to correlate with the degree of genetic divergence within each serotype. The H5-Con vaccine induced the greatest levels of protection against the divergent H5N1 influenza strains. However, this serotype has the least amount of genetic divergence (~5%). This would be logical as the various influenza strains would be less divergent from the vaccine gene. On the other hand, the H1-Con vaccine appears to induce greater levels of immunity as compared to the H3-Con vaccine. While both the H1 and H3 serotypes have greater levels of genetic diversity between strains, as compared to the H5 serotype, there is more genetic divergence in the H1 serotype than the H3 serotype, ~21% and ~14%, respectively. A more exhaustive analysis using more mouse-adapted influenza viruses may better elucidate the correlation between genetic diversity and the efficacy of centralized vaccine genes. However, only these two H3 mouse-adapted influenza viruses were available for this study.

In general, mice vaccinated at the highest dose of vaccine do not show any signs of disease. This may be due primarily to antibody titers that are high enough to completely neutralize the virus prior to infection. However, the mice vaccinated with lower doses of vaccine do show signs of disease and weight loss when challenged with lethal influenza viruses. These data indicate that the primary infection is not circumvented solely due to sterilizing antibody responses. Anti-influenza T cell immunity as well as changes in the cytokine responses due to vaccination may play a significant role in the protective effects observed at the lower doses. T cell depletion and cytokine profile studies may provide important details into this mechanism of protection.

While the lower doses were less effective, it should be pointed out that the challenge viruses consisted of stringent doses that had 100-fold greater lethality than has ever been observed in humans (H5N1 = 58% mortality). Therefore the protection we observed at the low dose vaccine would likely be much more effective against nonlethal endemic and pandemic strains. Therefore these doses are very translatable to humans and indicate just how effective these vaccines can be against extremely pathogenic influenza viruses. Based on body weight, the intermediate vaccine dose of 5 X 10^7^ vp/mouse equates to ~1.5 X 10^11^ vp/human. This is a dose of Ad that is commonly used in human clinical trials and found to be safe and well tolerated [29–32]. Again, studies that evaluated the vaccines in the context of less stringent influenza challenges would most likely show an increase in vaccine efficacy at even lower doses. Another attractive feature of this study is the use of Ad type 4 as a vaccine vector. Ad type 4 is already used in the military to vaccinate recruits and prevent adenovirus-associated acute respiratory distress. It has been found to be safe and effective [22]. Our data clearly indicate that a centralized H5-Con gene could be used to protect against H5N1 influenza. First-responders, such as military recruits, would benefit greatly from this multivalent vaccine platform. These data show that centralized HA-Con genes can be used to generate broadly reactive immune responses against divergent H1, H3 and H5 influenza virus strains and show promise for translation into human vaccines.

## Supporting Information

S1 Fig HeLa cells were transduced with replication-defective E1 deleted Ad5 viruses expressing the consensus genes. A hemagglutination assay was performed by serially diluting the cells in DPBS in a U bottom assay plate. Fifty microliters of a 1.0% solution chicken red blood cells was added and incubated at room-temperature for 1 hour. A control virus expressing the eGFP-Lucifease expression gene was included as a control for the detection of possible Ad-specific agglutination.
(TIFF)

S2 Fig The transmembrane domains were predicted using the TMAP software available publicly through the San Diego Super Computer Biology Workbench program. (TIFF)

S3 Fig A box shade alignment of the consensus protein sequences was performed using ClustalW. (TIFF)
