# Supplementary figures and images for: Centralized Consensus Hemagglutinin Genes Induce Protective Immunity against H1, H3 and H5 Influenza Viruses
Source: PLoS One. 2015 Oct 15;10(10):e0140702. doi: 10.1371/journal.pone.0140702 (PMC4607479; doi:10.1371/journal.pone.0140702)

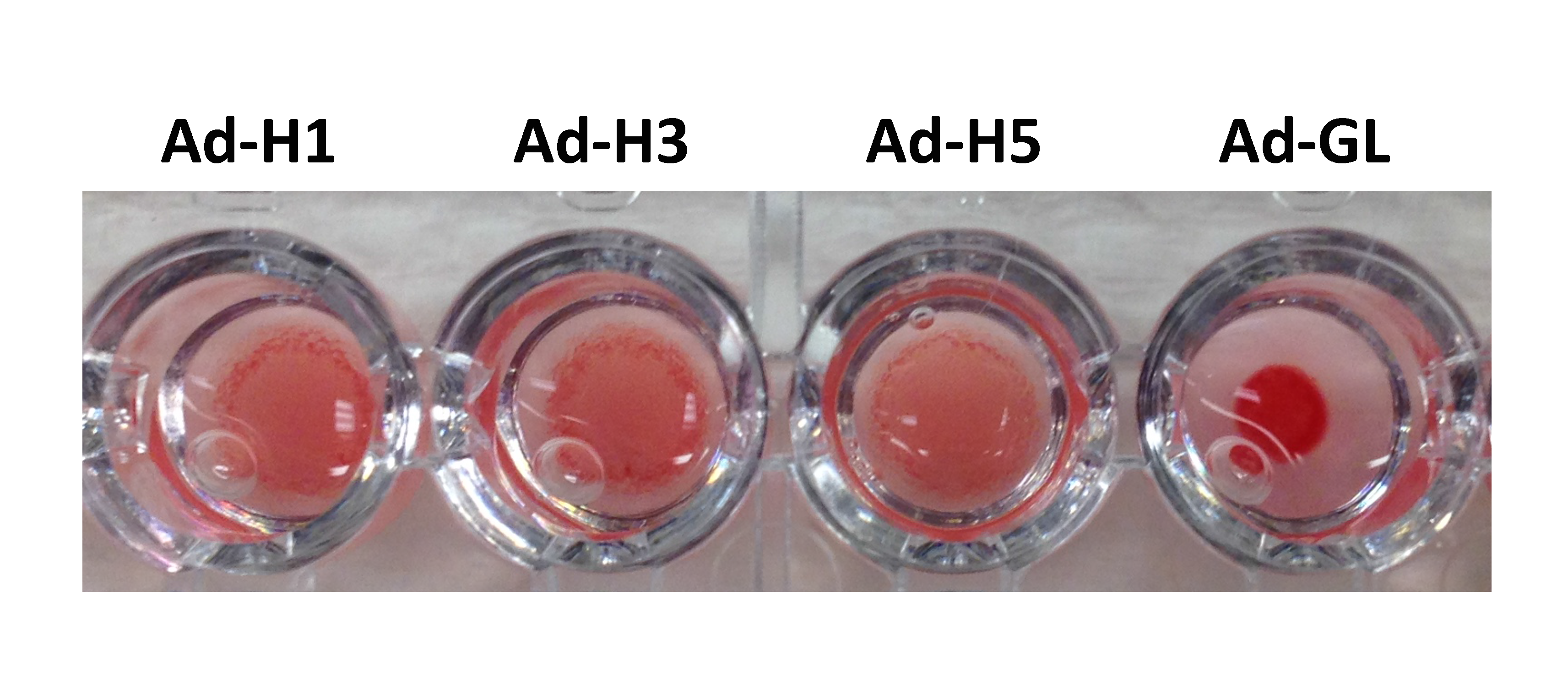

Supplement: S1 Fig — A hemagglutination assay was performed by serially diluting the cells in DPBS in a U bottom assay plate. Fifty microliters of a 1.0% solution chicken red blood cells was added and incubated at room-temperature for 1 hour. A control virus expressing the eGFP-Lucifease expression gene was included as a control for the detection of possible Ad-specific agglutination. (TIFF) [file pone.0140702.s001.tiff]

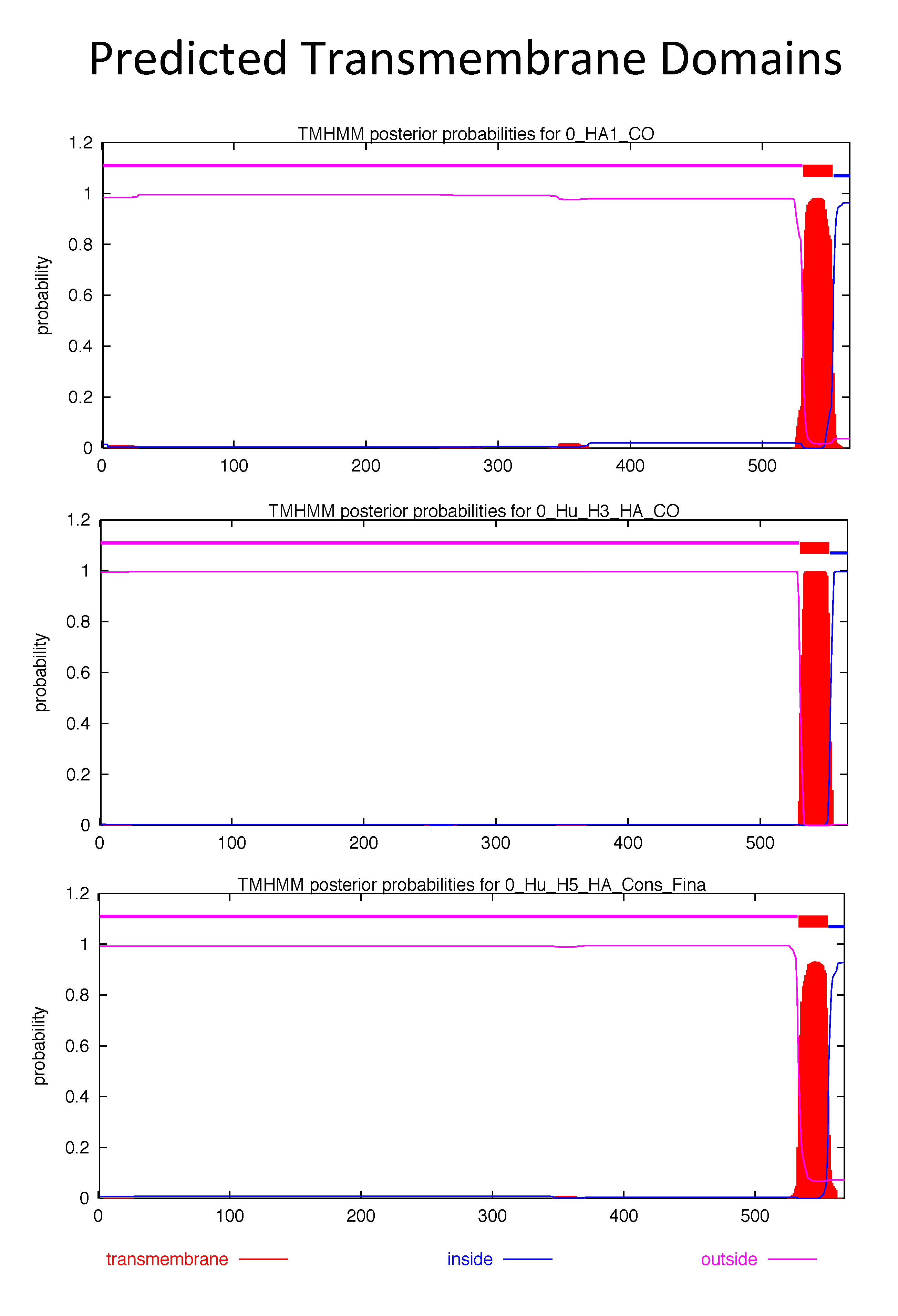

Supplement: S2 Fig — (TIFF) [file pone.0140702.s002.tiff]

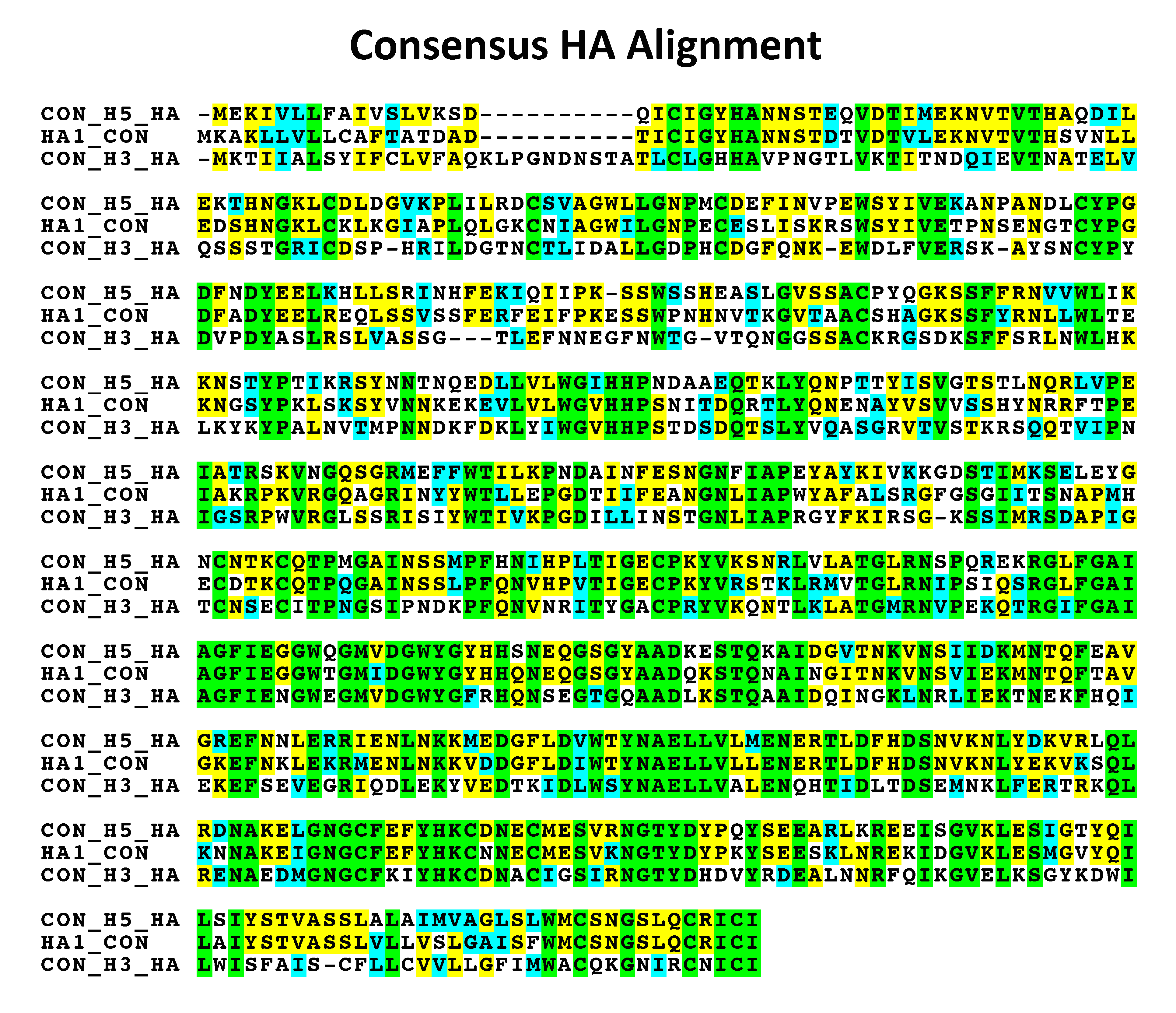

Supplement: S3 Fig — (TIFF) [file pone.0140702.s003.tiff]
